# Peripheral Glial Cells in the Development of Diabetic Neuropathy

**DOI:** 10.3389/fneur.2018.00268

**Published:** 2018-05-02

**Authors:** Nádia Pereira Gonçalves, Christian Bjerggaard Vægter, Lone Tjener Pallesen

**Affiliations:** ^1^Department of Biomedicine, Nordic-EMBL Partnership for Molecular Medicine, Danish Research Institute of Translational Neuroscience (DANDRITE), Aarhus University, Aarhus, Denmark; ^2^The International Diabetic Neuropathy Consortium (IDNC), Aarhus University, Aarhus, Denmark

**Keywords:** diabetic neuropathy, satellite glial cells, Schwann cells, peripheral nervous system, dorsal root ganglion, diabetes mellitus

## Abstract

The global prevalence of diabetes is rapidly increasing, affecting more than half a billion individuals within the next few years. As diabetes negatively affects several physiological systems, this dramatic increase represents not only impaired quality of life on the individual level but also a huge socioeconomic challenge. One of the physiological consequences affecting up to half of diabetic patients is the progressive deterioration of the peripheral nervous system, resulting in spontaneous pain and eventually loss of sensory function, motor weakness, and organ dysfunctions. Despite intense research on the consequences of hyperglycemia on nerve functions, the biological mechanisms underlying diabetic neuropathy are still largely unknown, and treatment options lacking. Research has mainly focused directly on the neuronal component, presumably from the perspective that this is the functional signal-transmitting unit of the nerve. However, it is noteworthy that each single peripheral sensory neuron is intimately associated with numerous glial cells; the neuronal soma is completely enclosed by satellite glial cells and the length of the longest axons covered by at least 1,000 Schwann cells. The glial cells are vital for the neuron, but very little is still known about these cells in general and especially how they respond to diabetes in terms of altered neuronal support. We will discuss current knowledge of peripheral glial cells and argue that increased research in these cells is imperative for a better understanding of the mechanisms underlying diabetic neuropathy.

## Introduction

Peripheral neuropathy is a disorder affecting the peripheral sensory and autonomic nerves as a consequence of trauma or disease. The most common cause of peripheral neuropathy in the United States and Europe is type 2 diabetes, affecting 30–50% of diabetic patients (1–3). The global prevalence of diabetic neuropathy (DN) can be appreciated by the fact that an estimated 415 million people worldwide aged 20–79 had diabetes in 2015, and this number is expected to grow to 640 million by 2040, predominantly as a result of the increasing prevalence of type 2 diabetes. Furthermore, 318 million adults are estimated to have impaired glucose tolerance, or pre-diabetes, giving them a high risk of developing the disease (4).

In the peripheral nervous system (PNS), diabetes may induce several kinds of neuropathies. The most common type is known as the “stocking and glove” distribution of neuropathy, i.e., a bilateral and symmetric damage to nerves of the feet and hands (5). Patients commonly experience sensory impairment, which involves either loss of sensation or spontaneous feelings of touch, vibration, pricking, and hot and cold pain. The condition may also be accompanied by hypersensitivity (6, 7). Later, in the course of the disease, motor weakness and multiple organ dysfunction may be observed, arising from affected motor and autonomic nerves, respectively (8, 9). Unfortunately, no effective treatments for DN have been developed, and the main clinical strategy is still glucose control. However, a 2012 Cochrane review of all available clinical studies revealed that even rigorous glucose control is only able to decrease the incidence of DN in type 1 diabetes, whereas little to no effect was observed in type 2 diabetes (10), emphasizing the continuous need for more research.

The development of targeted therapies for DN has been hindered by a lack of understanding of the complex and different etiologies of this disorder. A number of molecular mechanisms have been proposed, ranging from metabolic to vascular hypotheses, with components of inflammation. These approaches have certainly contributed to our understanding of the various pathophysiological processes occurring in peripheral neurons under diabetic conditions. However, they have offered little help in preventing or reversing the symptoms of DN. It is noteworthy that the vast majority of clinical and basic research in DN has focused on the neuronal component of the nerves, presumably from the perspective that the neurons are the functional signal-transmitting cells. However, substantial amounts of data from research in the development and regeneration of the PNS categorically define the glial cells as indispensable components when it comes to maintaining neuronal structure and function as well as nourishing the axons and promoting survival and growth upon injury. Obviously, there is a huge gap in our understanding of how PNS glial cells respond to the diabetic conditions, and how this affects their important roles of nourishing and promoting neuronal survival and health. In this review, we address the evolving concepts relating PNS glial cells, namely satellite glial cells (SGCs) and Schwann cells, to diabetic neuropathogenesis based on insights from *in vitro* studies, animal models, and patients.

## Satellite Glial Cells in Diabetic Neuropathy (DN)

### Satellite Glial Cells—An Introduction

**Figure 1:**
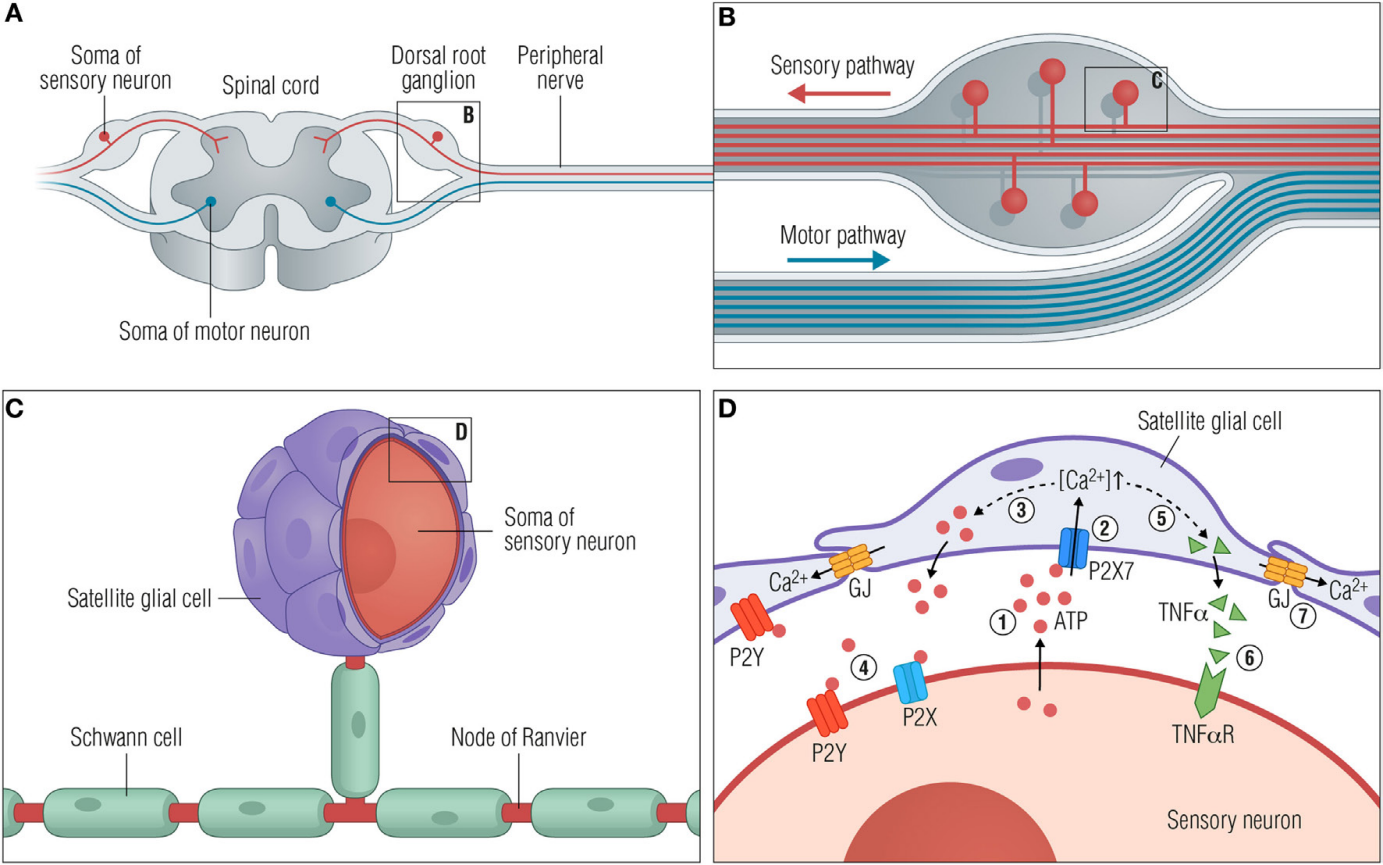
Satellite glial cells respond to neuronal stress. **(A)** A peripheral ganglion exemplified by the dorsal root ganglion (DRG). **(B)** Organization of the DRG. The pseudounipolar DRG neurons branch off with a peripheral process that connects with target tissues in the periphery and a central process in the spinal cord dorsal horn. **(C)** Close-up view of a single neuronal soma with myelinating Schwann cells along the axons and satellite glial cells (SGCs) closely enwrapping the soma. **(D)** Schematic diagram showing the communication between SGCs and the soma of the sensory neurons following neuronal stress. The consequences of injury or stress are various changes, some of which are outlined here. (1) ATP is released from the soma to the extracellular space. (2) Secreted ATP activates purinergic receptors such as P2X7 on the SGCs, causing an increase in [Ca^2+^]_in_. (3) The higher [Ca^2+^]_in_ leads to greater release of ATP from SGCs (dashed line), further increasing the extracellular ATP concentration and subsequently (4) activation of P2X and P2Y receptors on the neurons. (5) Activation of P2X7 receptors furthermore facilitates the release of cytokines such as TNFα (dashed line) from the SGC, which then (6) binds and activates neuronal TNFα receptors. (7) Activated SGCs are characterized by an upregulation of connexin channels and thereby an increase in gap junctions and intercellular coupling of SGCs by Ca^2+^ waves. Abbreviations: GJ, gap junctions; TNFα, tumor necrosis factor α; TNFαR, TNFα receptor.

Satellite glial cells are localized in the sensory and autonomic ganglia of the PNS, forming a thin and tight sheath around each individual neuronal soma (Figure 1). The number of SCGs enclosing a neuron is proportional to the size of the neuronal soma and is in the range of 4–10 SGCs per neuron in mice and 8–12 SGCs per neuron in rats (11), while the numbers in humans are still to be determined. The SGCs surrounding a single soma are connected and communicate *via* gap junctions, forming a distinct functional unit (12). An extremely narrow gap of 20 nm between the neuronal soma and the SGCs enables tight control of the neuronal extracellular space by both the neuron and the SGCs. Evidence for bidirectional communication between neurons and SGCs is found in the sensory ganglia (13–15), largely mediated by purinergic P2 receptors, but our understanding of this communication is still limited.

The SGCs are flattened cells, and the intimate spatial relationship between SGCs and neurons complicates their isolation and analysis. Consequently, the biology of SGCs is inadequately characterized under normal as well as pathological conditions. However, in recent decades, an increasing amount of research utilizing rodent models of nerve injury and dorsal root ganglion (DRG) explant assays have established SGCs as homeostatic cells providing support and modulation of sensory neurons; it is now clear that SGCs express various neurotransmitter receptors, transporters, and ion channels, allowing them to monitor neuronal activity and homeostasis. Furthermore, they release neurotransmitters and neuroactive substances such as ATP and cytokines as part of a glia-to-neuron signaling (12, 14, 16–20).

### SGCs Respond to Neuronal Stress

It has become evident that a wide variety of neuronal stress situations trigger a state of activation in the SGCs. This was demonstrated in animal models of traumatic nerve injury (21–24), but also in animal models of diabetes (25–27), inflammation (28, 29), chemotherapy (30), and herpes simplex infection (31). Altogether, these findings strongly suggest that SGC activation is a normal physiological response to neuronal stress.

Activated SGCs are characterized by profound changes [for extensive reviews, see Ref. (17, 32, 33)] that generally appear as cell proliferation (22), upregulation of glial fibrillary acidic protein (GFAP) (34, 35), and upregulation of connexin channels (17, 36, 37). Injured or stressed neurons have been shown to release ATP, targeting SGC purinergic P2 receptors (13, 38) and thereby initiating gap junction-dependent Ca^2+^ waves between the SGCs (17, 39). The principal purinergic receptors expressed in SGCs are the metabotropic P2Y receptor subtypes 1, 2, 4, 6, 12, and 13 (20, 40), and the ionotropic P2X7 receptor in both rodents (41, 42) and human DRGs (43).

Interestingly, SGC-to-neuron signaling is mediated by the activation of SGC P2X7 receptors, resulting in the secretion of both tumor necrosis factor α (TNFα) and ATP from SGC (44) and contributing to hyperexcitability of the enwrapped neurons (41, 44–46). Furthermore, recent publications demonstrate how SGCs surrounding neighboring sensory neurons respond to nerve injury by inducing neuronal activity coupling, arguing that neuronal stress may spread within a ganglion (23, 45, 47). In accordance with these observations, it has also been shown how the blocking of gap junctions attenuates hyperexcitability of DRG neurons and mechanical hyperalgesia (48).

These and other findings establish SGCs as important components for neuronal homeostasis and activity and show that SGC activation is a normal consequence of neuronal stress and injury. It is, therefore, a realistic perspective that targeting SGCs is a feasible strategy for modulating neuronal activity and obtain improved pain management and neuronal regeneration in patients suffering from various peripheral neuropathies.

### A Perspective on SGC Involvement in DN

Characteristics of DN are nerve dysfunction and degeneration of nerve endings in the extremities, likely due to a complex combination of factors such as metabolic imbalances, inflammation, hyperglycemia, oxidative stress, and lower oxygen tension (9, 49). Analyses of DRGs indeed show that the DRG neurons are susceptible to injury by metabolic and hypoxic stressors in models of diabetes, as the neurons show altered gene expression of, e.g., voltage-gated calcium channels and sodium channels, increased neuronal excitability as well as mitochondrial dysfunction leading to excess formation of reactive oxygen species, deregulation of Ca^2+^ homeostasis, and Ca^2+^ signaling (9, 50–54).

As outlined above, SGCs respond to a wide range of neuronal stress situations, but only very few reports on SGCs in a diabetic context have been published. Hanani et al. described how SGCs are activated in a streptozotocin-induced type 1 diabetes model in rodents, revealed by a fourfold increase in the number of neurons surrounded by GFAP-positive SGCs in the mouse DRG and a fivefold increase in rats (25). In line with this, it was recently found that preventing P2X7 receptor upregulation in a rat diabetic model inhibited the activation of SGCs as evaluated by GFAP expression in the DRG (26). Moreover, inhibiting upregulation of P2X7 receptors reduced the release of TNFα, thereby inhibiting the excitability of DRG neurons and reducing mechanical and thermal hyperalgesia in diabetic rats.

Further support for SGC responsiveness came from the observation that streptozotocin-induced diabetes in mice substantially enhanced DRG neuron expression and activity of pyruvate dehydrogenase kinases (PDK2 and PDK4), key regulatory enzymes in glucose metabolism (27). This upregulation seemed necessary to induce SGC reactivity, as PDK2/4 deficiency significantly attenuates SGC GFAP immunoreactivity following streptozotocin-treatment. The authors indicated that PDK2/4 may be expressed by activated SGCs; however, the presented data are not sufficiently convincing.

Together, these data establish that SGCs indeed respond to the diabetes-induced neuronal stress and, therefore, may participate in the generation and maintenance of DN. It may, however, also be that increased glucose acts upon the SGCs directly, initiating SGC reactivity without prior neuronal stress. Such a view is supported by the observation that aldose reductase is expressed by SGCs but not neurons in the DRG of both normal and diabetic rats (55). As described in further detail in the Section “Schwann Cells in DN” below, increased flux through the polyol pathway converts glucose to sorbitol by aldose reductase, increasing cellular stress by increasing osmolarity and depleting cellular stores of nicotinamide adenine dinucleotide phosphate (NADPH). However, in contrast to Schwann cells, the role of the polyol pathway in SGCs in relation to DN is completely unexplored.

Unfortunately, it has proven very difficult to isolate and study the SGCs due to their extremely close spatial relationship with the neurons. Attempts to study SGCs are made using primary cultures of isolated SGC for *in vitro* studies (56, 57). However, it has been reported how mouse trigeminal SGCs rapidly change morphology and gene expression in culture when they lose their tight association with the neuronal soma (58). This is likely a general feature also applying to DRG and autonomic SGCs, complicating both *in vitro* identification of SGCs and a meaningful interpretation of results.

To overcome these obstacles, we have recently developed a protocol that allows complete dissociation and isolation of highly pure SGCs by fluorescence-activated cell sorting (FACS) using SGC-specific antibodies. The method further allows purification of high-quality RNA from the fixed and permeabilized cells (59), and proteomic data can also be obtained. We believe that this strategy will initiate a deeper investigation of SGCs at transcriptional and translational levels *in vivo*, increasing our knowledge on SGC biology and their role in the complex pathobiology underlying DN.

## Schwann Cells in DN

### Pathological Hallmarks

**Figure 2:**
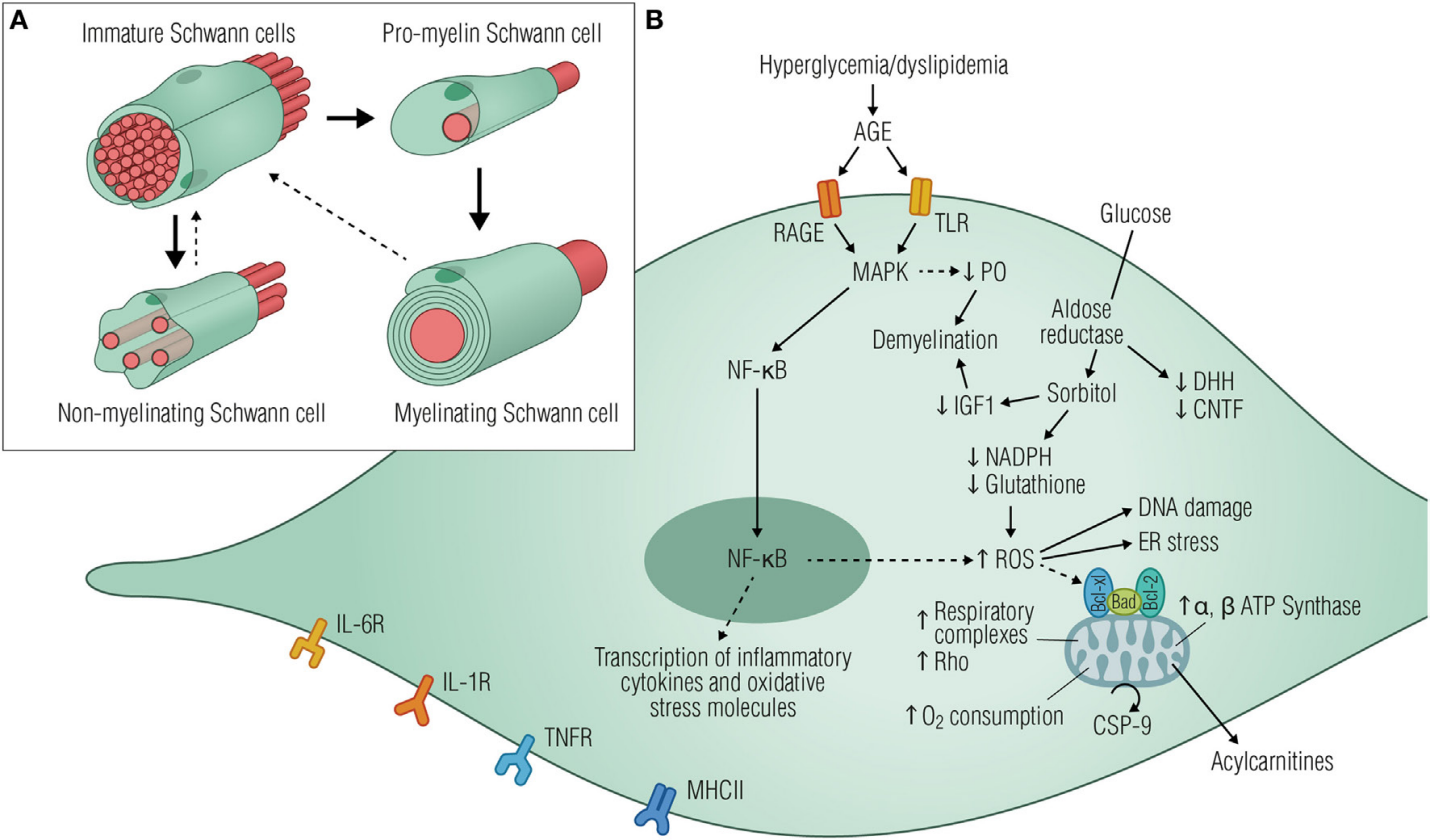
Diabetic stressors lead to Schwann cell dysfunction and neurodegeneration. **(A)** Schematic illustration of the main developmental transitions of Schwann cells. Dashed arrows denote the reversibility of the final stage, where mature myelinating and non-myelinating cells might dedifferentiate as a result of injury or disease. **(B)** Elevated glucose levels are shunted into the polyol pathway by aldose reductase, depleting cytosolic NADH, and ultimately reducing regeneration of glutathione, an important cellular antioxidant. Consequently, glucose metabolism generates local oxidative damage, reduces the production of neurotrophic factors, activates the ER stress, and causes DNA damage, all of which drives the cells to an immature phenotype. Signs of mitochondrial stress triggered by hyperglycemia are also evident in Schwann cells by the increased expression of ATP synthase subunits and respiratory chain complexes I, III, IV, and V, thus increasing oxygen consumption and apoptotic signaling events. As a result, acylcarnitines are released from stressed Schwann cells, leading to axonal degeneration and fiber loss. AGE activation of RAGE and/or TLR induces downstream signaling events mediated, at least in part, by the activation of MAPK and NF-κB with consequent transcription of inflammatory cytokines and oxidative stress molecules. Schwann cells have been recognized as immune-competent cells, expressing MHC class II molecules and several cytokine receptors such as IL-6R, IL-1R, and TNFR. Since MHC II antigen reactivity was detected in Schwann cells from patients suffering from diabetic neuropathy, it is plausible that these cells might be functioning as antigen processing and presenting cells contributing to immune responses within the peripheral nerves. Abbreviations: AGE, advanced glycation end products; RAGE, receptor for advanced glycation end products; TLRS, toll-like receptors; MAPK, mitogen-activated protein kinase; P0, protein 0; NF-κB, nuclear factor κB; NADPH, nicotinamide adenine dinucleotide phosphate-oxidase; IGF1, insulin-like growth factor 1; ROS, reactive oxygen species; ER, endoplasmic reticulum; CSP-9, caspase-9; IL-6R, interleukin-6 receptor; IL-1R, IL-1 receptor; TNFR, tumor necrosis factor receptor; MHCII, major histocompatibility complex II.

Schwann cells are the most abundant glial cells of the PNS, ensheathing all axons of peripheral nerves as either myelinating or non-myelinating cells (Figure 2A). Schwann cells are not merely passive insulators of axons but dynamic partners with the potential to modulate neuronal biology by providing metabolic support (60), enrichment of sodium channels (61), as well as modulating responses to tissue injury (62, 63). In disease states such as diabetes, Schwann cell function might be disturbed, compromising glial–axon communication, and nerve homeostasis, ultimately leading to fiber loss, neurodegeneration, and pain. Signs of Schwann cell stress are well demonstrated by unspecific morphological changes found in nerve preparations from humans as well as cat and rodent models of disease (64–67), indicating compromised Schwann cell functionality. As pathology proceeds, mild segmental axonal demyelination and remyelination begin to occur in the presence of a normal axon (68, 69), suggesting that Schwannopathy may very well underlie the primary damage to nerve fibers and be the first step in the pathogenesis of DN.

We will now review metabolic pathways known to be activated in Schwann cells during DN (summarized in Figure 2B), with an emphasis on the concept that Schwannopathy might be a central mechanism that leads to PNS damage in diabetes.

### Revisiting the Polyol Pathway

Hyperglycemia-driven increased flux through the polyol pathway is the most studied pathogenic mechanism of DN. Excess glucose is converted to sorbitol by aldose reductase, increasing cellular osmolarity and depleting stores of NADPH, an important component for the generation of nitric oxide and restoration of the crucial antioxidant glutathione. As a result, oxidative stress will contribute to cellular damage and dysfunction, impairing nerve structure and homeostasis (9) (Figure 2B). Strikingly, in the endoneurium, aldose reductase is essentially expressed by myelinating Schwann cells (55), implying that increased flux through aldose reductase is an essential factor in hyperglycemic toxicity to these cells. Although diabetic rodents only show demyelination after long-term neuropathy or when in the presence of hypertension (70), there is nevertheless evidence of Schwann cell alterations in these models such as reduced production of myelin proteins (71) and neurotrophic factors like CNTF (72), NGF, and NT-3 (73, 74). Reduced desert hedgehog (DHH) expression by the Schwann cells further contributes to nerve fiber loss and conduction velocity impairment, which together with induced endothelial dysfunction contribute to the development of DN (75, 76).

Perturbations in Schwann cell metabolism will affect glial–axon communication and promote direct deleterious effects on neuronal function, resulting from myelin disruption, impaired regeneration, and slower nerve conduction velocity (49). One hypothesis, based on cell culture studies, is that hyperglycemia leads to sorbitol accumulation and reduces the expression of insulin-like growth factor 1, promoting Schwann cell dedifferentiation into an immature phenotype (77). Other studies highlight the reduced expression of glycoprotein P0, myelin basic protein, and myelin-associated glycoprotein by the Schwann cells as main culprits for myelin loss (78, 79). Reduced P0, in particular, was shown to be dependent on Schwann cell upregulation of the mitogen-activated protein kinase (MAPK) signaling, which in turn contributes to keeping the cell in a dedifferentiated state (79), creating a pathogenic feedback loop (Figure 2B).

Preclinical tests with aldose reductase inhibitors became the hot topic in the 1990s. In spite of promising results in the animal models (80), all human clinical trials failed, possibly due to poor trial design, incorrect dose, limited drug potency, or the inability of the compounds to cross the blood–nerve barrier (81), pointing toward the importance of new lines of research.

### Diabetic Stressors Increase Schwann Cell Dysfunction and Death

When cultured in an hyperglycemic environment, Schwann cells change their spindle-shaped morphology and look thin and short, with less extended cell bodies and failed process outgrowth (79, 82), consistent with glucose-triggered apoptotic signaling events such as cleavage of caspase-3 and DNA fragmentation (83).

High glucose-induced death in Schwann cells *in vitro* was associated with altered expression of caspase-9, BAX, and Bcl-2 (84), thus indicating mitochondrial internal stress (Figure 2B). In fact, mitochondrial dysfunction has been associated with the pathogenesis of DN in multiple ways: data from primary sensory neurons associated mitochondrial dysfunction with perturbed calcium homeostasis (85, 86). More recently, studies have demonstrated how mitochondrial deficits in Schwann cells seem to activate a maladaptive stress response, causing accumulation of acylcarnitines, which ultimately induced axonal degeneration upon release (87, 88).

Hyperglycemia has also been associated with remodeling of the Schwann cell mitochondrial proteome, with increased expression of α and β subunits of ATP synthase. Other consequences of high glucose are suboptimal respiratory capacity triggered by the increased overall rate of oxygen consumption, thus suggesting deficient oxidative phosphorylation in Schwann cells (89) (Figure 2B). Proteomic and metabolomic data from the peripheral nerve of a rodent model of type 1 diabetes showed upregulation of mitochondrial respiratory complexes and Rho GTPase 1. The fact that no similar differences were found in sensory or trigeminal ganglia assign the described changes to a disrupted Schwann cell mitochondrial function (90) and is believed to engage the glial demyelination program (91). Indeed, ultrastructural abnormalities in Schwann cell mitochondria are present in peripheral nerves from both human diabetic patients and animal models of disease (92, 93). Hence, targeting Schwann cell mitotoxicity might offer new insights into the therapeutics of DN by preventing demyelination and increasing axonal survival.

### Activation of an Immune-Like Phenotype in Schwann Cells

The non-enzymatic glycation of proteins leading to their dysfunction is a long-established feature of diabetes that has been linked to neuropathy. In recent years, this concept has been expanded to include pathological signaling of advanced glycation end products through its receptor (RAGE), which is located on axons and Schwann cells of the peripheral nerve (94). In fact, RAGE overexpression was evident in Schwann cells of the sural nerve from diabetic patients, possibly linking oxidative modification and inflammation with preclinical features of disease (95). The induced downstream events resulting from RAGE signaling involve activation of MAPKs and nuclear factor κB, with consequent transcription of inflammatory intermediates, production of ROS, and vasoconstriction (96) (Figure 2B). This scenario is similar to that of traumatic nerve injury where Schwann cells dedifferentiate and adapt a repair-mediating phenotype essential for myelin degradation and nerve regeneration (97).

However, with continuous exposure to deleterious signals such as hyperglycemia and modified lipoproteins, a feed-forward loop of injury is created due to enhanced immune responses, increased cellular oxidative, endoplasmic, and nitrosative stress (98–100). Schwann cells have also been recognized as immune-competent cells, as they express major histocompatibility complex II molecules and several toll-like and inflammatory receptors (Figure 2B) and produce several cytokines known to be involved in the pathogenesis of DN (101–105). As inflammatory cytokines can sensitize Aδ and C-fibers, these observations highlight the potential involvement of Schwann cells in the development of painful DN. In addition, inflammatory mediators produced by Schwann cells may contribute to the recruitment of immune cells like macrophages and T cells, which in turn can induce Schwann cell damage by triggering apoptosis (106–108). Overall, a deeper investigation of the Schwann cell inflammatory response might offer new insights into the stressful cellular mechanisms contributing to glial cell dysfunction, disruption of neuronal metabolism, axonal transport, or repair capabilities in DN.

## Conclusion

Despite the increasing knowledge into the importance of glial cells for nerve tissue function and homeostasis, most research on DN is focused on the peripheral sensory neurons. Significantly less is known about the peripheral glial cells, and particularly, the SGCs have hardly been touched in a diabetic context. Important insights into the fundamental biology of PNS glia have been obtained in models of nerve injury and inflammation, and we believe that many aspects of glia responsiveness can be extrapolated into other disease states such as diabetes mellitus. Future work should focus on obtaining a comprehensive understanding of the genetic and molecular architecture of SGCs and Schwann cells, unraveling how they support neurons and engage in bidirectional communication to modulate neuronal activity. Knowledge on the pharmacological properties of these glia will help unravel novel mechanisms and targets for peripheral neuropathies such as DN.

## Author Contributions

NG, CV, and LP equally contributed to the writing of the manuscript.

## Conflict of Interest Statement

The authors declare that the research was conducted in the absence of any commercial or financial relationships that could be construed as a potential conflict of interest.
